# An *in vitro* assay for toxicity testing of *Clostridium perfringens* type C β-toxin

**DOI:** 10.3389/fimmu.2024.1373411

**Published:** 2024-04-05

**Authors:** Marieke Hoonakker, Afshin Zariri, Lisette de Brouwer, Dionne David, Anouska Borgman, Arjen Sloots

**Affiliations:** Department of Product Characterization and Formulation, Intravacc B.V., Bilthoven, Netherlands

**Keywords:** *Cl. perfringens* type C β toxin, *in vitro*, cell-based assay, alternatives, 3R

## Abstract

**Introduction:**

Veterinary vaccines against *Clostridium perfringens* type C need to be tested for absence of toxicity, as mandated by pharmacopoeias worldwide. This toxicity testing is required at multiple manufacturing steps and relies on outdated mouse tests that involve severe animal suffering. *Clostridium perfringens* type C produces several toxins of which the β-toxin is the primary component responsible for causing disease. Here, we describe the successful development of a new cell-based *in vitro* assay that can address the specific toxicity of the β-toxin.

**Methods:**

Development of the cell-based assay followed the principle of *in vitro* testing developed for *Cl. septicum* vaccines, which is based on Vero cells. We screened four cell lines and selected the THP-1 cell line, which was shown to be the most specific and sensitive for β-toxin activity, in combination with a commercially available method to determine cell viability (MTS assay) as a readout.

**Results:**

The current animal test is estimated to detect 100 – 1000-fold dilutions of the *Cl. perfringens* type C non-inactivated antigen. When tested with an active *Cl. perfringens* type C antigen preparation, derived from a commercial vaccine manufacturing process, our THP-1 cell-based assay was able to detect toxin activity from undiluted to over 10000-fold dilution, showing a linear range between approximately 1000- and 10000-fold dilutions. Assay specificity for the β-toxin was confirmed with neutralizing antibodies and lack of reaction to *Cl. perfringens* culture medium. In addition, assay parameters demonstrated good repeatability.

**Conclusions:**

Here, we have shown proof of concept for a THP-1 cell-based assay for toxicity testing of veterinary *Cl. perfringens* type C vaccines that is suitable for all vaccine production steps. This result represents a significant step towards the replacement of animal-based toxicity testing of this veterinary clostridial antigen. As a next step, assessment of the assay’s sensitivity and repeatability and validation of the method will have to be performed in a commercial manufacturing context in order to formally implement the assay in vaccine quality control.

## Introduction

*Clostridium perfringens* type C is a Gram-positive, spore-forming bacterium that can be found as a normal component of soil or decaying vegetation and it is a common bacterium in the intestinal tract of humans and other domestic animals. It is a well-known cause of food poisoning as a result of poor preparation or conservation. The spores formed by the bacterium can withstand cooking temperature which can result in the formation of infective colonies. *Cl. perfringens* type C isolates produce among others α-toxin, β-toxin, perfringolysin O (1) and enterotoxin (2). The bacterium causes necrotic enteritis mainly in piglets and sheep and food-borne *Cl. perfringens* type C can also cause necrotic enterocolitis in humans. Several lines of evidence demonstrate that the β-toxin is the major pathogenic component of the bacterium (1).

The β-toxin is a monomeric pore-forming protein of 34,861 Da (3), that is highly sensitive to thiol group reagents and proteases and can easily change into a non-toxic form (4). β-toxin is extremely toxic to porcine and human endothelial cells and causes acute cell death (5, 6). The toxin is considered to form cationic selective oligomer pores in cell membranes resulting in influx of Ca^2+^, Na^+^ and Cl^-^, causing swelling and lysis of cells (7). The β-toxin can bind to the P2X_7_ receptor (8), though recent evidence also suggest the involvement of pannexin (9) and CD31 (10) in β-toxin mediated cell lysis. THP-1 cells express both the P2X_7_ receptor (7) and CD31 (11) and die as a result of exposure to *Cl. perfringens* type C β-toxin (8, 11).

Administration of inactivated *Cl. perfringens* type C preparations containing β-toxin has been shown to dramatically reduce the frequency of necrotic enterocolitis in humans (12, 13). For livestock, vaccines based on inactivated, so-called toxoided, β-toxin are successfully used (14) and were shown to induce protective antibody responses (15). β-toxoid vaccines are often mixed with antigens of various other *Clostridium* species to form multivalent clostridial vaccines for veterinary use. Quality control testing of the β-toxoid vaccines starts directly after bacterial cultivation and is performed at several steps until the blending and filling of the final product (16). The testing always addresses the toxicity of the toxin itself, but fulfills different purposes, *i.e.* quantification of toxin activity before inactivation or testing for absence of toxicity after inactivation. Depending on the purpose and the manufacturing stage the toxicity tests may have different names. Due to the historic implementation of toxicity testing before 1950 and its codification in pharmacopoeias in many regions, a broad variety of names are used globally. The common denominator is that toxicity testing heavily relies on mouse-based tests, that use many animals and can inflict severe suffering to the animals involved. The potency of the final product is often quantified by the induction of antibodies in rabbits, with the neutralizing capacities of the induced antibodies assessed in naïve mice. All these tests all have lethal endpoints, for which the β-toxin is responsible (17), except for the rabbits, and inflict severe suffering.

In the tests described above for *Cl. perfringens* type C, the mice primarily serve as indicators for the presence of active toxin, similar to the tests used for quality control of other *Clostridium*-based vaccines. For example, vaccines containing *Cl. septicum* also require mouse tests for both toxicity and potency. This principle of using an indicator to detect the presence of active toxin can be used to replace the animal testing with alternative methodologies. In 2012, Redhead *et al*. (16) developed an *in vitro* cell-based assay as an alternative for the toxicity and antigenicity tests for *Cl. septicum*, using the Vero cell line as indicator for toxin activity. This assay has undergone a large series of formal and regulatory steps including a Biological Standardisation Programme (BSP) study (BSP130) run by the European Directorate for the Quality of Medicines & HealthCare (EDQM) in order to prepare its implementation in a GMP production environment. The Vero cell assay is the official method in the European monograph 07/2022:0364 on *Cl. septicum* vaccine for veterinary use from July 2022.

Here, we applied a similar strategy of utilizing a cell line as indicator for *Cl. perfringens* type C β-toxin activity in a non-inactivated antigen preparation. This term describes the filtrated culture supernatant derived from an industrial vaccine manufacturing process, containing the active β-toxin. Whereas the simple presence of β-toxin can be addressed by PCR or Western blotting, these methods cannot distinguish between the active and inactivated forms of the toxin. However, as β-toxin is a pore-forming toxin, mammalian cells are well suited for assessment of its toxic effects and could, therefore, serve as an alternative toxicity indicator to the mouse. The susceptibility of a wide range of cells, among others Vero cells and various immune cell lines including THP-1 and U937 has been evaluated elsewhere (7, 18). The β-toxin had no effect on the Vero cells but affected all immune cell lines of which the THP-1 and the U937 cell line were the most sensitive. More recently, these results were confirmed by a different research group that also identified endothelial cells as β-toxin sensitive (10).

In the present study, from a panel of four tested cell lines, we identified two cell lines (THP-1 and A10) that were sensitive to the *Cl. perfringens* type C non-inactivated antigen, evaluated a selection of readout methods and selected the THP-1 cell line in combination with the MTS method as the most appropriate for our purposes. We determined the sensitivity of the resulting assay for *Cl. perfringens* type C non-inactivated antigen and its specificity for β-toxin, using a neutralizing monoclonal antibody and an antitoxin preparation directed against *Cl. perfringens* type C β-toxin. Importantly, we also demonstrated the compatibility of our THP-1 cell-based assay with an inactivated β-toxoid preparation. Together, our results show proof of principle for a new *in vitro* cell-based assay allowing detection of β-toxin activity. Upon validation, the assay has the potential to replace all mouse-based toxicity testing performed during production of *Cl. perfringens* type C vaccines.

## Materials and methods

### Cell lines and culture conditions

**Figure 1:**
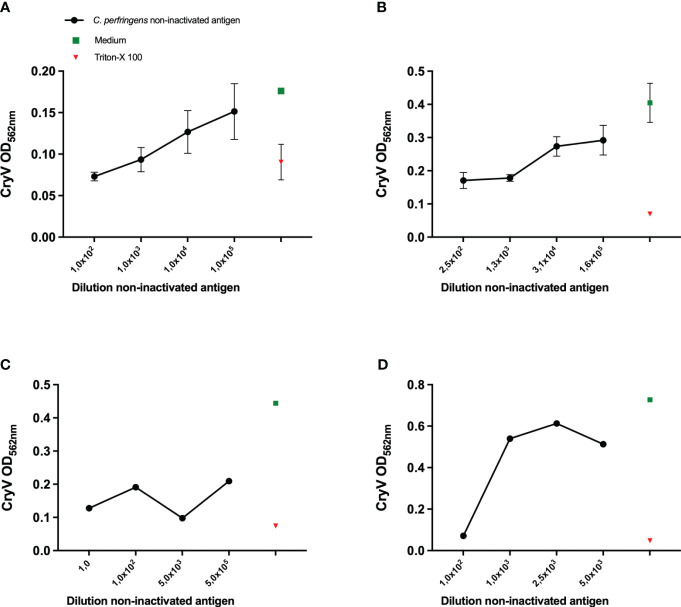
Sensitivity of various cell lines to *Cl. perfringens* type C non-inactivated antigen. THP-1 cells (PMA differentiated), **(A)**, A10 cells **(B)**, J774A.1 cells **(C)** and Vero cells **(D)** were exposed to the indicated dilutions of the *Cl. perfringens* type C non-inactivated antigen preparation, medium or Triton-X-100 for 16-24 hours. The loss of cells as a result of toxicity was measured by staining the remaining cells with Crystal Violet (CryV). Data are expressed as mean values of duplicate **(C, D)** or triplicate measurements ± SD **(A, B)**. Shown is one of three representative experiments **(B, D)** and one experiment **(A, C)**.

**Figure 2:**
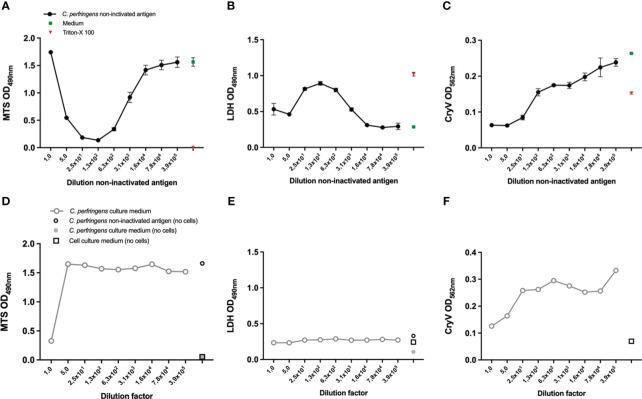
Selection of a suitable readout parameter for THP-1 and A10 cells and the effect of *Cl. perfringens* type C culture medium. THP-1 cells (PMA differentiated), **(A, B, D, E)** and A10 cells **(C, F)** were exposed to the indicated dilutions of the *Cl. perfringens* type C non-inactivated antigen preparation, *Cl. perfringens* type C culture medium, cell culture medium or Triton-X-100 for 16-24 hours. Cell viability was measured with the MTS assay **(A, D)** and the LDH assay **(B, E)**. The loss of cells as a result of toxicity was measured by staining the remaining cells with Crystal Violet (CryV) **(C, F)**. Data are expressed as mean values ± SD of triplicates **(A–C)** or shown as single measurements **(D–F)**. Shown is one of three representative experiments **(A, C)**, one out of two representative experiments **(B, D, E)** and data from a single experiment **(F)**.

**Figure 3:**
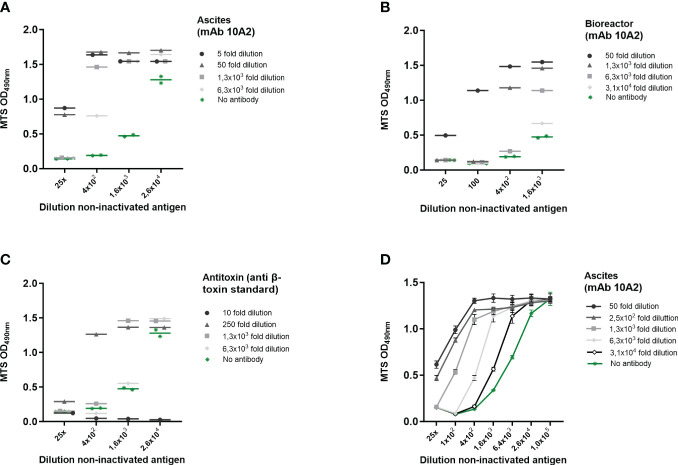
β-toxin specific induction of cell death on THP-1 cells. *Cl. perfringens* type C non-inactivated antigen preparation dilutions were incubated with the indicated dilutions of ascites-derived mAb 10A2 **(A, D)** and bioreactor-derived mAb 10A2 **(B)** and an international anti-β-toxin standard (2CPBETAAT) **(C)**. THP-1 cells (PMA differentiated) were exposed to the antibody-β-toxin mixtures for 16-24 hours. Cell viability was measured with the MTS assay. Data are expressed as single or duplicate measurements **(A–C)**, or as mean values ± SD of triplicates **(D)**. Shown is one of four experiments **(A)** and one experiment **(B–D)**.

**Figure 4:**
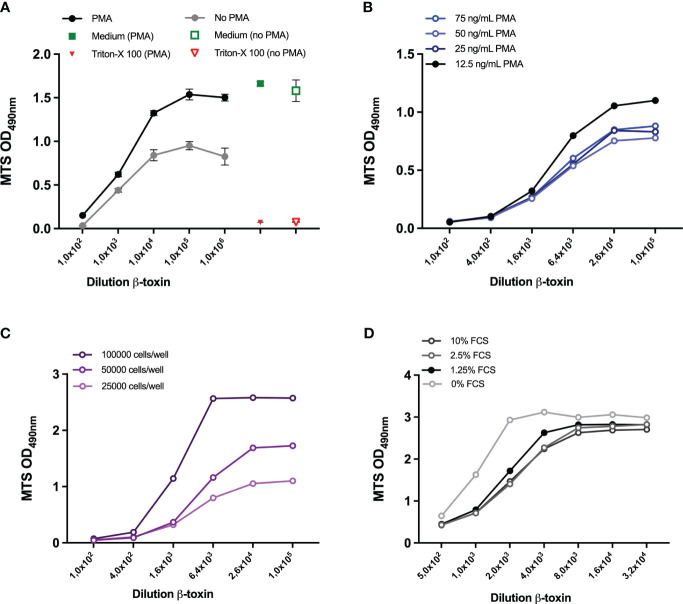
Optimization of cell density and PMA and FCS concentration. THP-1 cells (25000 cells/well) were either left untreated or differentiated with 300 ng/ml PMA **(A)** or 12.5, 25, 50, 75 ng/mL PMA **(B)** overnight. THP-1 cells at a density of 25000, 50000, 100000 **(C)** or 75000 **(D)** were seeded and differentiated with 12,5 ng/ml PMA overnight. Subsequently, all cells were exposed to the indicated dilutions of the β-toxin sample for 16-24 hours, either prepared in medium with 10% FCS **(A–C)** or FCS concentrations ranging between 0-10%. Subsequently, cell viability was assessed by MTS assay. Data are expressed as mean values ± SD of triplicates **(A)** or duplicate measurements **(B-D)**. Shown is one experiment for each condition examined.

**Figure 5:**
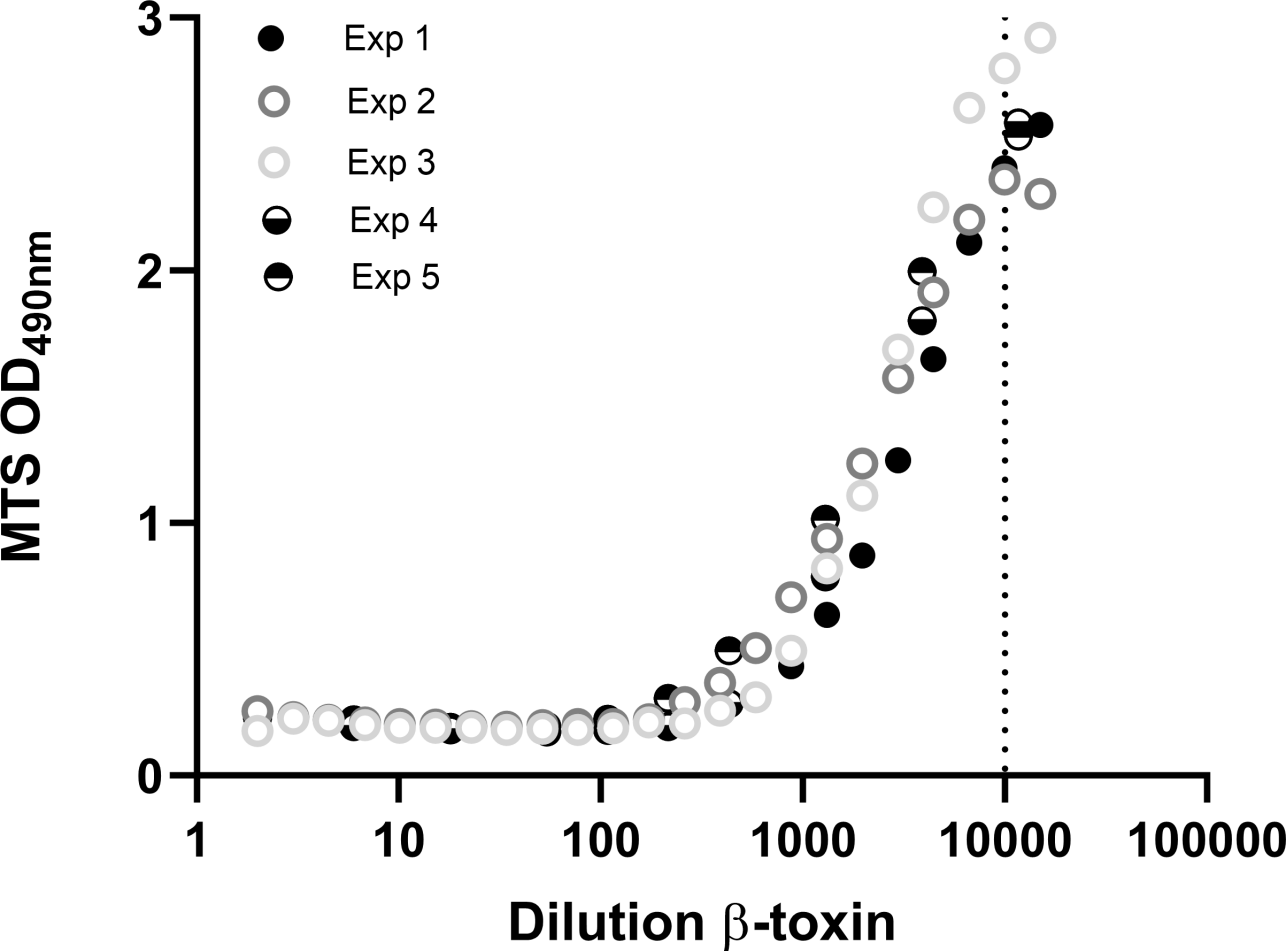
Repeatability of the THP-1-based toxicity assay. THP-1 cells (75000 cells per well) were differentiated with 12.5 ng/mL PMA overnight and exposed to indicated dilutions of the β-toxin for 16-24 hours, prepared in medium containing 0.5% FCS. Cell viability was measured with the MTS assay. Data are mean values of duplicate measurements (exp 1, 2, 4 and 5) or shown as single measurements (exp 3). Toxin data of experiment 4 and 5 are also shown in **Figure 6**.

**Figure 6:**
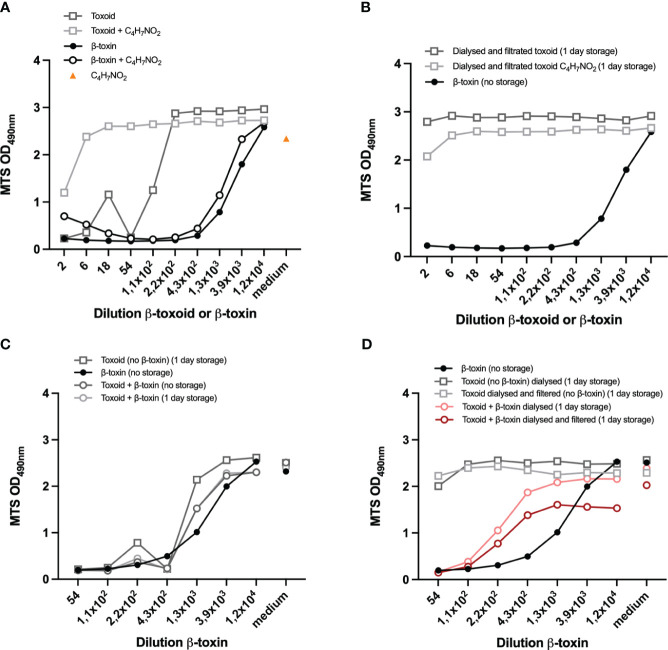
Compatibility of the assay with (spiked) β-toxoid. Preparations of toxoid and β-toxin were incubated with 25% acetoacetamide (C_4_H_7_NO_2_) **(A)** or dialyzed and filtrated (overnight) and incubated with 25% C_4_H_7_NO_2_ or left untreated **(B)**. Controls are shown in **Supplementary Figure S5A**. Toxoid (undiluted) was spiked with 1/54 dilution of the β-toxin or left untreated. Samples were prepared on the day of the experiment (no storage) or incubated overnight (1 day storage) as indicated **(B-D)**. Toxoid (undiluted) and non-inactivated antigen (1/54 dilution)-spiked toxoid samples were prepared and dialyzed overnight and filtrated as indicated **(B, D)**. Representative β-toxin and β-toxin-spiked toxoid samples were prepared on the day of the experiment **(B-D)**. THP-1 cells (75.000 cell per well) were differentiated with 12,5 ng/mL PMA overnight and subsequently exposed to the indicated samples for 16-24 hours, prepared in medium containing 0,5% FCS. In **A** and **B** the toxoid or β-toxin dilutions are indicated, while in **C** and **D** the β-toxin dilution is indicated and the corresponding toxoid concentrations are shown in **Supplementary Figures S5B, C**. Control measurements of cells incubated with medium with C_4_H_7_NO_2_ **(A)** or medium only **(C, D)** are indicated by “medium” on the x-axis. Cell viability was measured with the MTS assay. Datapoints are mean values of duplicate measurements from one experiment. A direct comparison between the two experiments shown in this figure is shown in **Supplementary Figure S5C**.

J774.1 cells (ATCC; TIB-67) were cultured in DMEM medium supplemented with 100 U/ml penicillin, 100 µg/ml streptomycin, 0.3 mg/ml L-glutamine (Gibco), and 10% FBS (Gibco). A10 cells were purchased at ATCC (CRL-1476) and were cultured in DMEM medium supplemented with 1 mM pyruvate (Gibco), 100 U/ml penicillin, 100 µg/ml streptomycin, 0.3 mg/ml L-glutamine (Gibco) and 10% FBS (Gibco). Vero cells were obtained from WHO (10–87) (originally derived from ATCC (CCL-81)) and were cultured in VP-SFM medium (Gibco), supplemented with 2 mM L-Glutamine (Gibco). THP-1 cells used in **Figures 1**, **2**, **3A–C**, **4A**, **Supplementary Figures S1**, and **S2** were obtained from an in-house stock. To confirm results obtained with this stock, subsequent experiments were performed with THP-1 cells from a stock derived from a vial purchased at ATCC (TIB-202) (**Figures 3D**, **4B–D**, **5**, **6**, **Supplementary Figures S3**–**S5**). THP-1 cells were maintained in RPMI medium (Gibco), supplemented with 50 µM β-mercaptoethanol, 100 U/ml penicillin, 100 µg/ml streptomycin, 0.3 mg/ml L-glutamine (Gibco) and 10% FBS (Gibco). All cell lines were cultivated at 37°C in a humidified atmosphere of 5% CO_2_.

### Cell seeding, exposure to *Cl. perfringens* type C non-inactivated antigen and readout methods

THP-1 cells were seeded at 25000 cells/well in a 96-well flat bottom plates (Greiner Bio One) and differentiated with 300 ng/mL PMA (Sigma), unless mentioned otherwise. A10 cells, J774.1 and Vero cells were seeded at 100000, 25000 and 25000 cells/well in 96-well flat bottom plates. For all cell types, viable cells were counted following staining with a 0.4% trypan blue solution and only cell populations with a minimal viability of 70% were used for seeding. After seeding, cells were incubated over night at 37°C in a humidified 5% CO_2_ incubator. Subsequently, the cells were exposed to various dilutions of the *Cl. perfringens* type C non-inactivated antigen preparation, containing active β-toxin (kindly provided by a pharmaceutical company that was part of the VAC2VAC consortium (http://www.vac2vac.eu), hereafter referred to as company B), medium or Triton-X-100 (Merck) for 16-24 hours. Cell viability was verified either by staining with 1) Crystal Violet (Sigma) - which stains living cells; 2) by addition of MTS (MTS assay, Promega) - a substance (3-(4,5-dimethylthiazol-2-yl)-5-(3-carboxymethoxyphenyl)-2-(4-sulfophenyl)-2H-tetrazolium) that is converted into colored formazan by living cells or 3) the LDH assay (Promega) performed on the culture supernatant, measuring the conversion of tetrazolium salt (iodonitrotetrazolium violet) into a red formazan product by cellular lactate dehydrogenase (LDH) released into the supernatant upon cell death. The MTS and LDH assay were performed according to the manufacturer’s protocol and the quantity of formazan product as measured after 3.5-4.5 hours for the MTS assay and 30 minutes for the LDH assay at 490 nm. Crystal Violet (Sigma) staining was also performed according to manufacturer’s protocol and was measured at 562 nm.

### Preparations of non-inactivated antigen and antibody-antigen mixtures

*Cl. perfringens* type C non-inactivated antigen samples were prepared in medium containing 10% FCS (unless mentioned otherwise) at the indicated dilutions within one hour before addition to the cells. To determine the specificity for β-toxin, the *Cl. perfringens* type C non-inactivated antigen samples were pre-incubated for 30 min incubation at 2-8°C on an orbital shaker (250 rpm) with dilutions of a neutralizing monoclonal antibody (clone 10A2 purchased from the USDA; concentration unknown, produced either in mice (referred to as ‘Ascites’) or in a bioreactor (referred to as ‘Bioreactor’)) or an international antitoxin standard (2CPBETAAT from NIBSC; 4770 IU of antitoxin, reconstituted in 1 mL PBS, referred to as ‘Antitoxin’).

### *Cl. perfringens* type C β-toxoid and β-toxoid spiked with β-toxin

*Cl. perfringens* type C toxoid (kindly provided by company B) samples were prepared in medium containing 0.5% FCS at the indicated dilutions within one hour before addition to the cells. To neutralize the effect of residual formaldehyde (CH_2_O) present in the inactivated toxoid preparation, the undiluted β-toxoid and toxin preparations were incubated with 25% acetoacetamide (C_4_H_7_NO_2_) for 30 minutes at room temperature or dialyzed against PBS overnight at 2-8°C. 0.5 g/L formaldehyde served as a control. If indicated, dialyzed samples were passed through a 0.22 µm filter before being added to the cells. For the spiking experiment, the undiluted toxoid preparation was treated with a 1/54 dilution of the *Cl. perfringens* type C non-inactivated antigen or left untreated. The toxoid or toxin samples were prepared on the day of the experiment or incubated overnight as indicated.

### Statistical analysis

As the dose-response curves of β-toxin dilutions (x-axis) versus the OD at 490 nm (y-axis) follow a symmetrical sigmoidal shape, 4-parameter curve fitting (GraphPad Prism version 10.1.2) was used to mathematically describe the curves that were based on triplicate measurements (**Figures 3D**, **Supplementary Figure S5D**) and the curves shown in **Figure 5** (duplicate measurements and one single measurement). The linear part of **Figure 5** was analyzed using simple linear regression on the average values of duplicate measurements and one single measurement. The results are shown in **Supplementary Figure S4**.

## Results

### Sensitivity of cell lines to *Cl. Perfringens* type C non-inactivated antigen preparation

Inspired by the *in vitro* assay based on the Vero cell line in combination with Crystal Violet staining, which proved to be suitable for assessment of toxicity of *Cl. septicum* vaccines and intermediate products (16), we aimed at using this strategy as the readout for the current *Cl. perfringens* type C toxicity test, as well. From a wider range of cell lines that was evaluated elsewhere (7, 10, 18), we selected THP-1 cells as one of our candidate cell lines, along with the β-toxin sensitive cell line A10 and the P2X_7_ expressing murine J774A.1 cell line (19). Vero cells were included as a control. Both THP-1 and A10 cells (**Figures 1A, B**) were sensitive to *Cl. perfringens* type C non-inactivated antigen in a concentration-dependent manner, though the measured ODs were relatively low. The measured ODs for the Crystal Violet staining of J774A.1 (**Figure 1C**) were also very low and not concentration-dependent. Whereas the ODs for the Crystal Violet staining were higher for Vero cells (**Figure 1D**), toxicity was only observed for a 100-fold dilution of the *Cl. perfringens* type C non-inactivated antigen preparation or less diluted, suggesting this cell line has a lower sensitivity compared to the THP-1 and A10 cell lines. THP-1 cells and A10 cells were therefore selected for subsequent experiments.

### Selection of a suitable readout parameter to assess either cell death or viability

In **Figure 1**, the remaining cells after *Cl. perfringens* type C non-inactivated antigen exposure were visualized by staining with Crystal Violet as a readout for viability, like the Vero-cell based test for veterinary *Cl. septicum* vaccines. However, since the measured ODs were relatively low the MTS and the LDH assays were evaluated as alternative methods to estimate cell viability. The MTS assay measures the conversion of the MTS tetrazolium compound into formazan by living cells. The assay is therefore directly proportional to the number of living cells. The LDH assay measures the activity of lactate dehydrogenase, a stable enzyme that is released upon cell lysis. Released LDH converts the tetrazolium salt into formazan and is therefore proportional to the number of dying cells. Exposure of THP-1 cells to *Cl. perfringens* type C non-inactivated antigen in a dilution range between 25 to 3.9x10^5^ fold resulted in a concentration dependent increase of the MTS signal (**Figure 2A**) and decrease of the LDH signal (**Figure 2B**). The range of the ODs was acceptable for both readout parameters and substantially higher than the Crystal Violet readout (**Figure 1A**) with the dynamic range (highest OD/lowest OD) being 12-fold for MTS and 3.2-fold for LDH (**Figures 2A, B**). Exposure of THP-1 cells to a one- or five-fold dilution of the *Cl. perfringens* type C non-inactivated antigen preparation resulted in a higher signal of the MTS assay as compared to the 25-fold dilution (**Figure 2A**), which most likely is the result of a direct reaction between the *Cl. perfringens* type C non-inactivated antigen preparation and the MTS reagent (**Figure 2D**). Remarkably, exposure of THP-1 cells to the one- and five-fold dilutions of the *Cl. perfringens* type C non-inactivated antigen preparation was associated with a lower LDH signal as compared to the 25-fold dilution, which cannot be attributed to the *Cl. perfringens* culture medium or a reaction of the preparation with the LDH reagent (**Figure 2E**), but nonetheless should be taken into account. More importantly, the absence of an effect of the *Cl. perfringens* culture medium (5-3.9x10^5^ fold dilution) on THP-1 viability indicates that the effect of the *Cl. perfringens* type C non-inactivated antigen can be specifically attributed to the β-toxin or other (toxic) components in the preparation. Exposure of A10 cells to the *Cl. perfringens* type C non-inactivated antigen preparation in the range of 10-3.9x10^5^ fold dilution resulted in a very slight concentration dependent increase in MTS signal with a dynamic range of 1.5-fold (**Supplementary Figure S1A**). The level of LDH released by A10 cells was only affected by a 10-fold dilution of the non-inactivated antigen preparation to the point of total cell death (Triton-X-100 control), while all other dilutions (50-3.9x10^5^) resulted in the release of LDH at a level of the medium control (**Supplementary Figure S1B**). In contrast the Crystal Violet staining does suggest a toxin concentration dependent effect with a dynamic range of 3.8 (**Figure 2C**), however the effect is not specific since the *Cl. perfringens* type C culture medium has a similar effect (**Figure 2F**). Consequently, the THP-1 cells in combination with the MTS assay were selected as most promising for further assay development.

### Determination of assay specificity using antibodies

To further determine whether the toxicity can be specifically attributed to β-toxin activity, experiments were performed where THP-1 cells were exposed to serially diluted *Cl. perfringens* type C non-inactivated antigen in the presence of serial dilutions of three different sources of β-toxin specific antibody preparations (ascites-derived and bioreactor-derived monoclonal antibody 10A2, and an international anti-β-toxin standard (2CPBETAAT)). All antibody preparations neutralized the effect of the *Cl. perfringens* type C non-inactivated antigen preparation, which is reflected in an increase in THP-1 viability (**Figures 3A-D**). The strength of neutralization differed between the three antibody preparations. Whereas mAb 10A2 from ascites had the highest neutralizing capacity, the international antitoxin clearly had the lowest neutralizing capability (**Supplementary Figure S2A**, **Figure 3D**). Furthermore, cell viability was not affected by any of the included dilutions of mAb 10A2 (both from ascites or bioreactor sources), while the international antitoxin at a one- and ten-fold dilution did negatively affect cell viability (**Supplementary Figure S2B**). Taken together, the results clearly show that the toxicity on THP-1 cells induced by the *Cl. perfringens* type C non-inactivated antigen can be specifically attributed to the β-toxin. Therefore, effects will subsequently be indicated as being β-toxin dependent.

### Optimization of several assay parameters

The experimental setup thus far consisted of 25000 cells per well, 300 ng/ml PMA and 10% FCS. Initial optimization experiments with and without PMA (**Figure 4A**) and with different concentrations of PMA (data not shown), confirmed the necessity of PMA-differentiation of the THP-1 cells for the assay and demonstrated that lower PMA concentrations (75 ng/mL) are associated with a higher maximum OD at 490 nm. Lowering the PMA concentration down to 12.5 ng/mL further increased the OD and the dynamic range to 21-fold (**Figure 4B**). Using a PMA concentration of 12.5 ng/mL, the OD at 490 nm also increased when the number of cells was increased up to 50000 and 100000 cells per well (**Figure 4C**), though the latter cell densities did change the shape of the curve. In a subsequent experiment, a cell density of 50000 and 75000 cell per well (in combination with a PMA concentration of 12,5 ng/ml and 10% FCS, **Supplementary Figure S3**) was examined and a density of 75000 cells per well resulted in the highest OD, with a 11-fold dynamic range (**Supplementary Figure S3B**). After determination of the optimal cell density (75000) and PMA concentration (12.5 ng/ml), several concentrations of FCS were compared to the 10% used thus far. Lowering the FCS concentrations only had a slight effect (**Figure 4D**), 0% FCS being associated with the highest OD but also with a higher background. 0% FCS also changed the shape of the curve and slightly decreased dynamic range to 9,1-fold (**Supplementary Figure S3B**). Based on these results and personal communication with company B, 0.5% was chosen as the most optimal FCS concentration and used for sample preparation in subsequent experiments, while cell culturing was still performed in 10% FCS.

### Repeatability and sensitivity

Repeatability is an important aspect of toxicity tests and quality control tests in general and was evaluated here based on five individual experiments in which very similar dilution ranges of β-toxin were used (**Figure 5**). There is only a very small variability between the five experiments regarding the absolute maximal OD at 490 nm. The fold change between total toxicity (lowest OD) and no toxicity (highest OD) for all experiments was between 12 and 16 fold and the lowest dilution still affecting cell viability, indicating the assay’s sensitivity, was around 10000-fold dilution of the toxin in this R&D setting. Analysis of the data of the five experiments shown in **Figure 5** revealed that all adequately fit a four parameter logistic curve with similar r^2^ (0.98-0.99) and a slight range in EC_50_ (2163-3843 fold dilution) (**Supplementary Figure S4A**). The results were confirmed by fitting the data of **Figure 3D** and **Supplementary Figure S5D** (EC_50_ of 7086 and 1343 and r^2^ of 0.99 and 0.99, respectively) (**Supplementary Figures S4B, C**). Simple linear regression analysis was performed on a part of the curve (875-6650 fold dilution) and revealed that the r^2^ was 0.87 (**Supplementary Figure S4D**). Whereas sensitivity of the assay was sufficient in all of these experiments, the range in EC_50_ highlights the needed for dedicated experiments to examine the linear range of the curve in more detail. These experiments should be a part of a (pre-)validation study.

### Suitability of the assay for (spiked) *Cl. perfringens* type C toxoid samples

The assay developed so far allows detection of *Cl. perfringens* type C β-toxin activity in a specific and sensitive manner. In addition to the evaluation of β-toxin activity, detection of β-toxin in the context of a toxoided antigen preparation is of key importance because it would allow the assay to be used for non-toxicity testing of inactivated *Cl. perfringens* type C vaccine intermediate and end-products. We therefore determined the compatibility of the assay with a toxoided *Cl. perfringens* type C antigen preparation (**Figures 6A-C**) and its ability to detect β-toxin in the presence of this toxoid preparation by spiking with a fixed concentration of toxin (1/54 dilution) (**Figure 6D**). One of the obstacles is the residual free formaldehyde in commercial toxoid preparations. The toxoid itself negatively affects cell viability and a 220x dilution is required to negate this effect (**Figure 6A**), an effect that can primarily be attributed to residual formaldehyde present in the toxoid preparation (**Supplementary Figure S5A**). Various substances were tested for their ability to neutralize this effect (NaHSO_3_, Bovine Serum Albumin, Tris, glycine), but only acetoacetamide and dialysis (**Figures 6A, B**) against PBS effectively neutralized the formaldehyde effect. Acetoacetamide has a slight effect on cell viability (**Figure 6A**), but a one in six dilution is sufficient to negate this effect. Dialysis increased the sample volume from 1 to 1.5 mL, which was reflected in the protein content that decreased from 1407 µg/ml to 1092 µg/ml, while filtration had a negligible effect (1086 µg/ml), suggesting a minimal loss of material. This was confirmed by spiking toxoid with a 1/54 dilution of the non-inactivated antigen. Without any treatment, a pilot experiment demonstrated that the effect of the toxoid with a 1/54 dilution of the non-inactivated antigen is similar to the effect of the β-toxin itself and a discrimination between the negative effect of the toxoid and β-toxin is therefore impossible (**Figure 6C**). However, dialyzed spiked toxoid preparations (with and without filtration) caused cell death (**Figure 6D**), though to a smaller extent than the toxin itself. This is most likely not ue to a loss in β-toxin activity overnight, as storage for one day had no effect on the activity of the toxin (**Supplementary Figure S5D**). More importantly, the experiment demonstrates that dialysis of the toxoid is a treatment that allows for the detection of β-toxin in the context of a β-toxoid preparation.

## Discussion

There is a strong incentive to replace the use of animals for batch release testing of biological medicinal products, especially vaccines. Replacing *in vivo* quality control tests for vaccine batch release is particularly appealing where the *in vivo* test is merely used as a readout for toxicity and where a laboratory rather than the target species is used. The multiple mouse tests performed throughout the production process of *Cl. perfringens* type C vaccines are a typical example of tests in which animals function as a readout for toxicity. These mouse tests are performed for testing of toxin activity and quantity before inactivation, for absence of toxicity after inactivation and to measure the neutralization capacities of antibodies induced in rabbits to assess the presence of crucial epitopes on the toxoided antigen (20). Although various cell lines have been described as susceptible to *Cl. perfringens* type C β-toxin (18), their suitability for development of a cell-based alternative *in vitro* method had not been explored to date.

The current mouse tests for inactivated *Cl. perfringens* type C antigen based-vaccines allow for quantification of β-toxin activity. Any alternative assay should therefore be quantitative and allow for validation according to the VICH guidelines for the “Validation of analytical procedures: Definition and Terminology” (21) and “Validation of analytical procedures: Methodology” (22). These guidelines require determination of the specificity, accuracy, precision, linearity, range and limit of quantitation. In this study, the THP-1 cell line was selected as the most β-toxin sensitive cell line as compared to A10, J774A.1 and Vero cells (**Figure 1**). In combination with the MTS assay, PMA differentiated THP-1 cells allow for the detection of up to 1:10000 dilution of the β-toxin preparation with a linear range between a 1000-fold and a 10000-fold dilution making this assay approach more sensitive than the currently performed *in vivo* test which have an estimated sensitivity range of 100 – 1000-fold dilutions of the *Cl. perfringens* type C non-inactivated antigen (VAC2VAC industry partners, personal communication). This sensitivity was confirmed in five repetitive experiments (**Figure 5**), which also documented the assay repeatability. Nevertheless, formal assessment of the assay’s sensitivity and repeatability needs to be addressed in a subsequent validation study or directly by manufacturers.

The specificity is the ability to unequivocally assess an analyte (here β-toxin) in the presence of components which may be present in the sample under study. Here, we used the *Cl. perfringens* type C non-inactivated antigen preparation of a commercial manufacturer, cleared from *Cl. perfringens* type C bacteria using a 0.2 µm filter. Apart from β-toxin and culture medium, *Cl. perfringens* type C also produces α-toxin, perfringolysin O and enterotoxin (1, 2). The *Cl. perfringens* type C culture medium had no effect on THP-1 cell viability and a monoclonal antibody as well as an international antitoxin standard specific for β-toxin substantially reduced the detrimental effect of the *Cl. perfringens* type C non-inactivated antigen preparation on cell viability (**Figure 3**). The ascites-derived mAb 10A2 was the most potent of the three tested antitoxin preparations and at a dilution of 1:100 of the *Cl. perfringens* type C non-inactivated antigen preparation, the pre-incubation with ascites-derived mAb 10A2 resulted in complete neutralization. Based on these results, we conclude that the assay is specific for *Cl. perfringens* type C β-toxin.

Our *in vitro* assay should not only allow for testing of β-toxin activity in the non-inactivated antigen preparation, it should also allow for testing of traces of toxin in inactivated (*i.e.* toxoided) antigen preparations. To achieve inactivation of *Cl. perfringens* type C preparations, formaldehyde is usually added in sufficient quantities before filtration (23), to ensure the reliable and complete killing of the bacteria and quantitative inactivation of the toxins. This, however, results in the presence of residual formaldehyde in β-toxoid preparations, which is most likely the cause of the negative effect of low toxoid dilutions on cell viability (**Figures 6A**, **S5A** and **S5C**). A 220-fold dilution of the toxoid preparation was required to minimize this detrimental effect. Fortunately, both incubation with acetoacetamide and exchange of the solvent in toxoid preparations with PBS by dialysis are methods that can overcome the impact of residual formaldehyde. Spiking of the toxoid preparation with a 1/54 dilution of the β-toxin in combination with dialysis, proved a suitable method to determine β-toxin activity in the presence of a representative concentration of toxoid (**Figure 6D**), though to a lesser extent than the toxin itself. The cause of this slight loss of toxin activity is unknown and needs further investigation. The results, however, provide an indication for the assay’s specificity for β-toxin in the presence of toxoid, although additional studies are required to confirm these results.

Here, we provide proof of principle for a THP-1 cell-based assay as a suitable and reliable *in vitro* alternative for assessing β-toxin activity in *Cl. perfringens* type C non-inactivated antigen and toxoided vaccines. The study shows that the assay is sensitive and specific, but requires validation before it can serve as a replacement for current mouse testing performed during *Cl. perfringens* type C vaccine production and batch release. Such validation studies should include evaluation of parameters specified by the VICH, among others by using blinded spiked samples. The studies performed by Redhead *et al.* (16) might serve as an example, involving a side-by-side evaluation of the new THP-1 cell-based assay and the historic *in vivo* methods. Hence, the THP-1 cell-based assay described in this study offers great potential as a convenient quantitative *in vitro* alternative to the mouse tests performed throughout *Cl. perfringens* type C vaccine production. Following validation, its implementation is expected to reduce costs, speed up the vaccine release process and avoid unnecessary animal suffering.

## Data availability statement

The raw data supporting the conclusions of this article will be made available by the authors, without undue reservation.

## Ethics statement

Ethical approval was not required for the studies on humans in accordance with the local legislation and institutional requirements because only commercially available established cell lines were used. Ethical approval was not required for the studies on animals in accordance with the local legislation and institutional requirements because only commercially available established cell lines were used.

## Author contributions

MH: Data curation, Investigation, Methodology, Supervision, Writing – original draft, Writing – review & editing, Formal analysis, Visualization. AZ: Data curation, Formal analysis, Investigation, Methodology, Supervision, Writing – original draft, Writing – review & editing. LB: Investigation, Methodology, Writing – original draft, Writing – review & editing. DD: Investigation, Methodology, Writing – original draft, Writing – review & editing. AB: Investigation, Methodology, Writing – original draft, Writing – review & editing. AS: Investigation, Methodology, Writing – original draft, Writing – review & editing, Conceptualization, Data curation, Supervision.

## Supplementary material

Supplementary Figure S1 Selection of a suitable readout parameter for A10 cells. A10 cells **(A, B)** were exposed to the indicated dilutions of the *Cl. perfringens* non-inactivated antigen sample, medium or Triton-X-100 for 16-24 hours. Cell viability was measured with the MTS assay **(A)** and the LDH assay **(B)**. Shown is data from one experiment **(A, B)**.

Supplementary Figure S2 Specificity of the assay for β-toxin. Dilutions of the *Cl. perfringens* type C non-inactivated antigen preparation were incubated with the indicated dilutions of ascites and bioreactor-derived mAb 10A2 and antitoxin 2CPBETAAT **(A)**. THP-1 cells (PMA differentiated) were exposed to the antibody-antigen mixtures **(A)** or only the antibodies from three different sources **(B)** for 16-24 hours and cell viability was measured with the MTS assay. The effects of the antibodies on the MTS reagent (no cells) were also measured for the highest concentrations of the antibodies **(B)**. Data are expressed as single measurements **(A, B)** in a single experiment **(A, B)**.

Supplementary Figure S3 Optimization of cell density and FCS concentration. THP-1 cells at a concentration of 50.000 **(A)** or 75.000 **(B)** per well were seeded and differentiated with 12,5 ng/ml PMA overnight. Subsequently, all cells were exposed to the indicated dilutions of the β-toxin sample for 16-24 hours, prepared in medium with FCS concentrations ranging between 2,5-10%. Subsequently, cell viability was assessed by MTS assay. Data are expressed as mean values of duplicate measurements from a single experiment **(A, B)**.

Supplementary Figure S4 Four parameter curve fitting and linear regression analysis of dose-response relationship. Statistical analysis was performed on the data of **(A)**, **(B, C)** and **(D)** as described in the materials and methods section.

Supplementary Figure S5 The effect of toxoid and formaldehyde (CH_2_O) on cell viability of neighboring wells, the effect of toxin-spiked toxoid preparations, a comparison between two experiments that included toxoid and the effect of one day storage on β-toxin activity. **(A)** 0,5g/L CH_2_O was diluted as indicated in medium containing 0,5% FCS. **(B)** Toxoid (undiluted) was spiked with 1/54 dilution of the β-toxin or left untreated, β-toxin preparations were prepared starting with a 1/54 dilution. Samples were prepared on the day of the experiment or incubated overnight as indicated. **(C)** Toxoid (starting at a 1/2 dilution) and toxin (starting at a 1/54 dilution) were prepared and dialyzed or dialyzed overnight and filtrated as indicated for two experiments. **(D)** Toxin samples were prepared at the indicated dilutions one day before the experiment (indicated as “1 day storage”) or on the day of the experiment (indicated as “no storage”). THP-1 cells (75.000 cell per well) were differentiated with 12,5 ng/mL PMA overnight and subsequently exposed to the indicated samples for 16-24 hours, prepared in medium containing 0,5% FCS. In **A** the dilution of CH_2_O is shown, while in **B** and **C** the toxoid dilutions are shown, the corresponding β-toxin dilutions are shown in **Figure 6C**. Cell viability was measured with the MTS assay. Data are mean values of duplicates for one experiment **(A–C)** or triplicates ± SD **(D)**.
