# Supplementary figures and images for: An in vitro assay for toxicity testing of Clostridium perfringens type C β-toxin
Source: Front Immunol. 2024 Apr 5;15:1373411. doi: 10.3389/fimmu.2024.1373411 (PMC11026656; doi:10.3389/fimmu.2024.1373411)

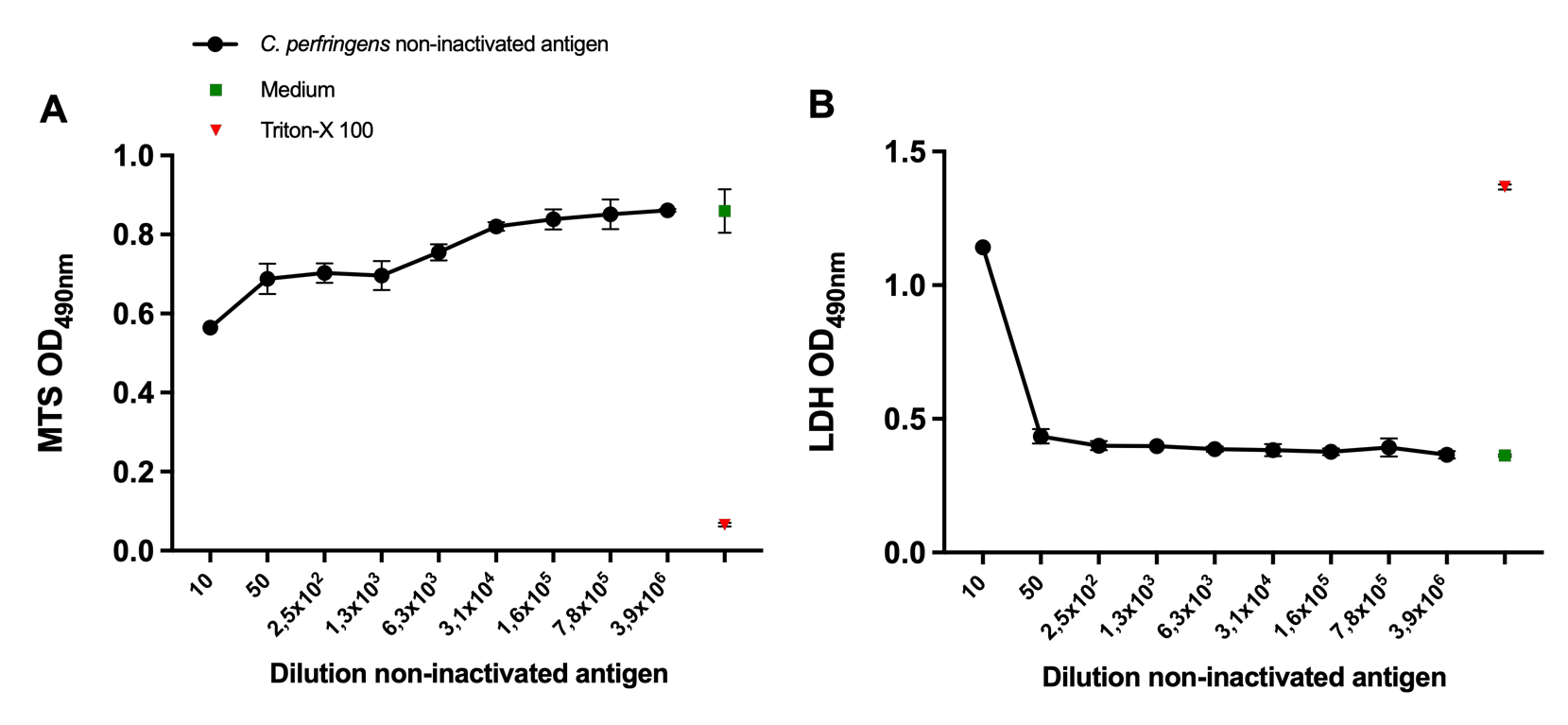

Supplement: Supplementary Figure S1 — Selection of a suitable readout parameter for A10 cells. A10 cells (A, B) were exposed to the indicated dilutions of the Cl. perfringens non-inactivated antigen sample, medium or Triton-X-100 for 16-24 hours. Cell viability was measured with the MTS assay (A) and the LDH assay (B). Shown is data from one experiment (A, B). [file Image_1.tiff]

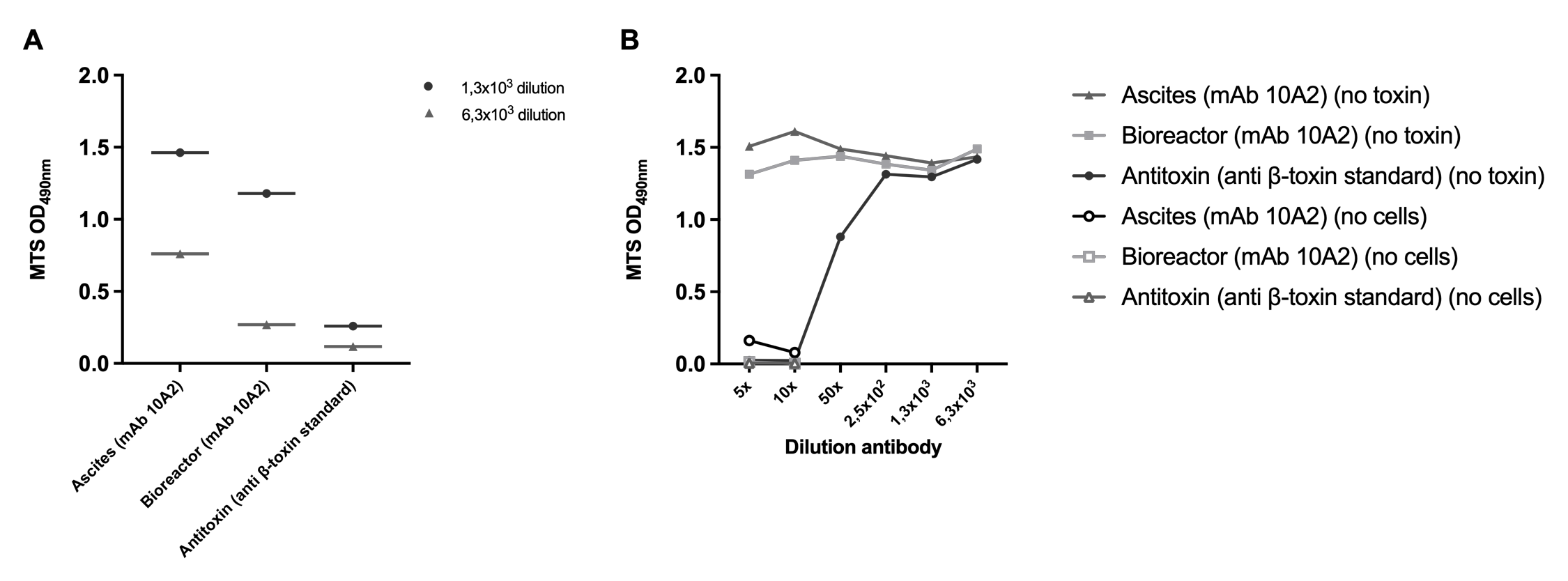

Supplement: Supplementary Figure S2 — Specificity of the assay for β-toxin. Dilutions of the Cl. perfringens type C non-inactivated antigen preparation were incubated with the indicated dilutions of ascites and bioreactor-derived mAb 10A2 and antitoxin 2CPBETAAT (A). THP-1 cells (PMA differentiated) were exposed to the antibody-antigen mixtures (A) or only the antibodies from three different sources (B) for 16-24 hours and cell viability was measured with the MTS assay. The effects of the antibodies on the MTS reagent (no cells) were also measured for the highest concentrations of the antibodies (B). Data are expressed as single measurements (A, B) in a single experiment (A, B). [file Image_2.tiff]

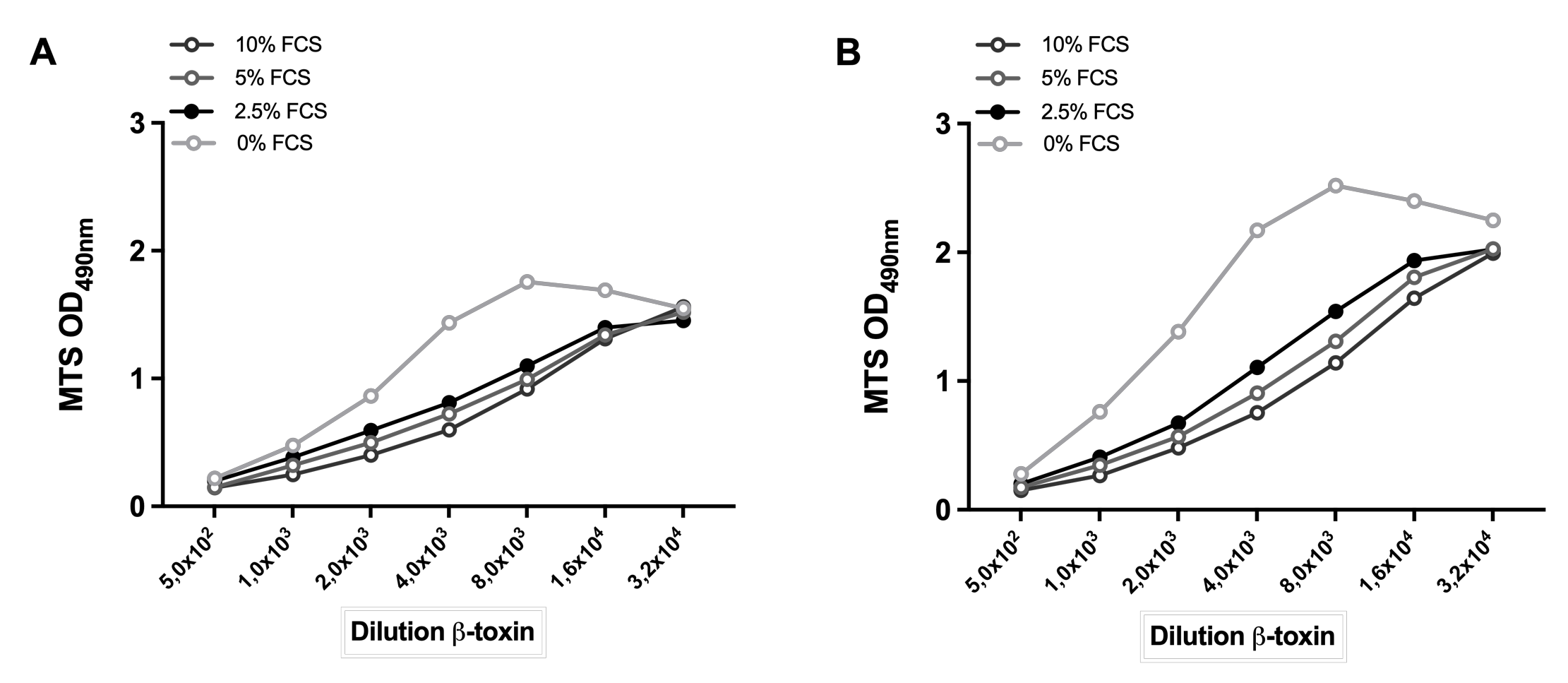

Supplement: Supplementary Figure S3 — Optimization of cell density and FCS concentration. THP-1 cells at a concentration of 50.000 (A) or 75.000 (B) per well were seeded and differentiated with 12,5 ng/ml PMA overnight. Subsequently, all cells were exposed to the indicated dilutions of the β-toxin sample for 16-24 hours, prepared in medium with FCS concentrations ranging between 2,5-10%. Subsequently, cell viability was assessed by MTS assay. Data are expressed as mean values of duplicate measurements from a single experiment (A, B). [file Image_3.tiff]

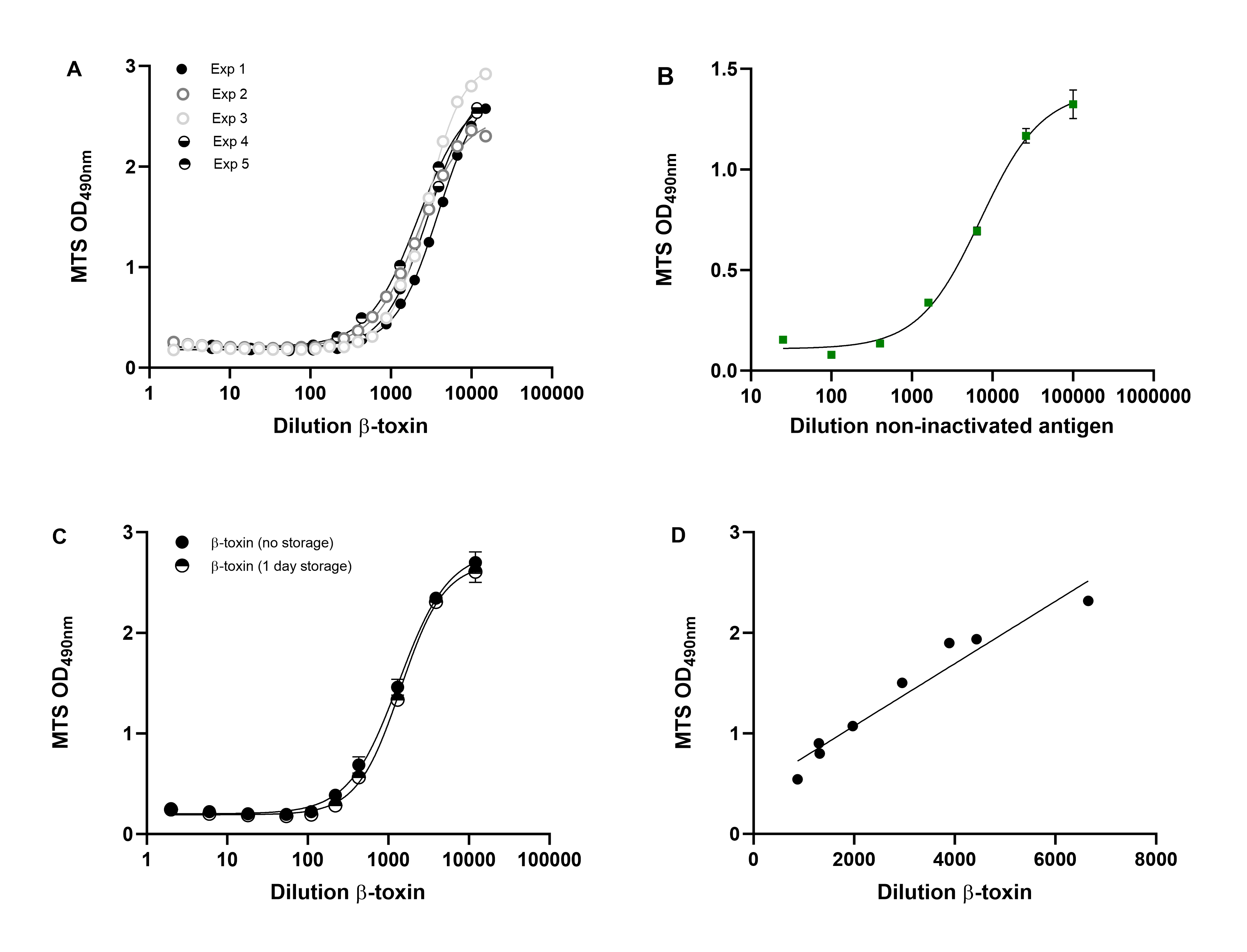

Supplement: Supplementary Figure S4 — Four parameter curve fitting and linear regression analysis of dose-response relationship. Statistical analysis was performed on the data of (A), (B, C) and (D) as described in the materials and methods section. [file Image_4.tif]

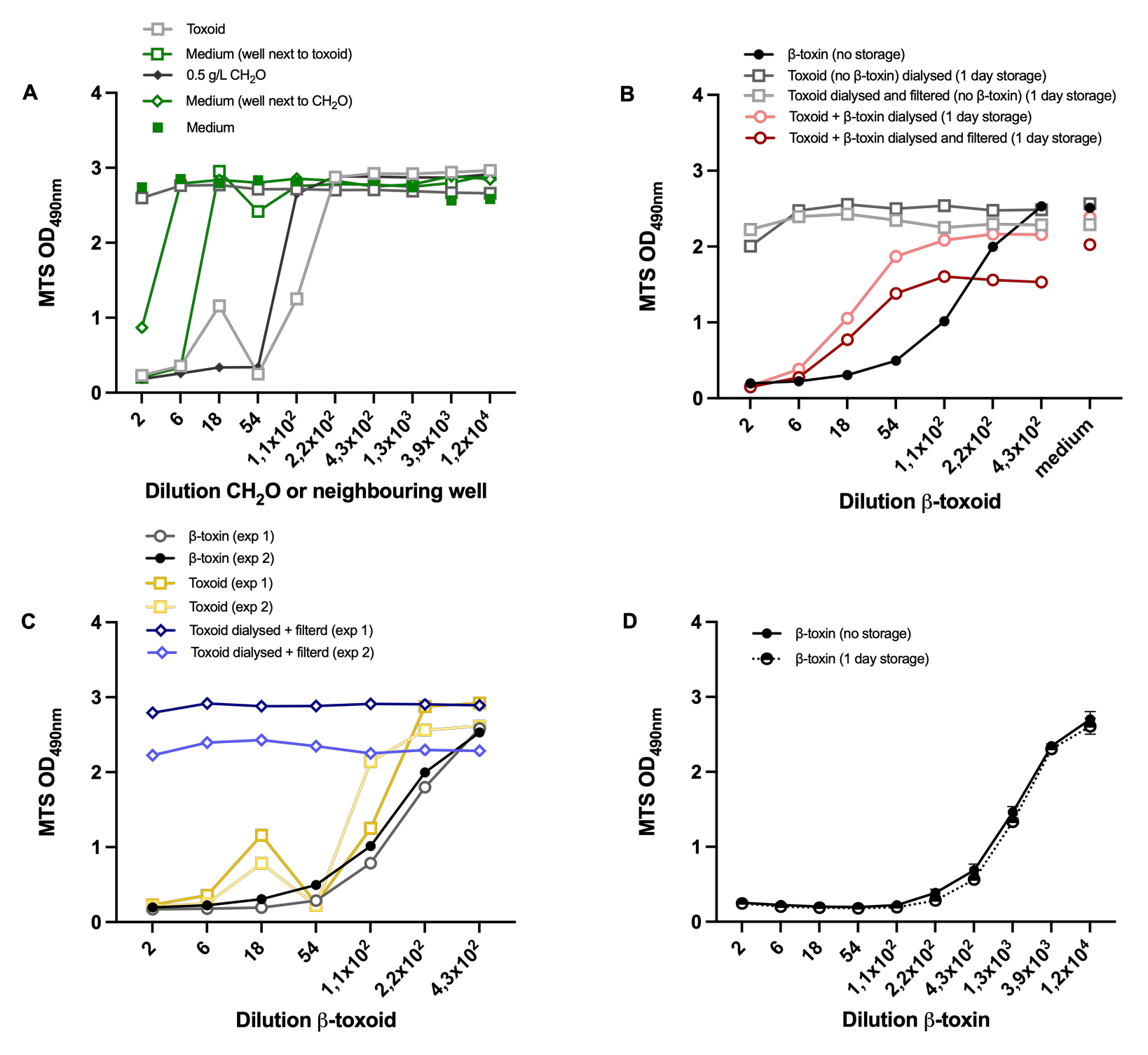

Supplement: Supplementary Figure S5 — The effect of toxoid and formaldehyde (CH2O) on cell viability of neighboring wells, the effect of toxin-spiked toxoid preparations, a comparison between two experiments that included toxoid and the effect of one day storage on β-toxin activity. (A) 0,5g/L CH2O was diluted as indicated in medium containing 0,5% FCS. (B) Toxoid (undiluted) was spiked with 1/54 dilution of the β-toxin or left untreated, β-toxin preparations were prepared starting with a 1/54 dilution. Samples were prepared on the day of the experiment or incubated overnight as indicated. (C) Toxoid (starting at a 1/2 dilution) and toxin (starting at a 1/54 dilution) were prepared and dialyzed or dialyzed overnight and filtrated as indicated for two experiments. (D) Toxin samples were prepared at the indicated dilutions one day before the experiment (indicated as “1 day storage”) or on the day of the experiment (indicated as “no storage”). THP-1 cells (75.000 cell per well) were differentiated with 12,5 ng/mL PMA overnight and subsequently exposed to the indicated samples for 16-24 hours, prepared in medium containing 0,5% FCS. In A the dilution of CH2O is shown, while in B and C the toxoid dilutions are shown, the corresponding β-toxin dilutions are shown in Figure 6C . Cell viability was measured with the MTS assay. Data are mean values of duplicates for one experiment (A–C) or triplicates ± SD (D). [file Image_5.tiff]
